# Inflammation of the Perodontal Membrane, Periodontitis, Alveola Abscess, Necrosis, and Other Sequelæ

**Published:** 1899-06

**Authors:** R. Denison Pedley


ARTICLE II.

### INFLAMMATION OF THE PERIODONTAL MEMBRANE, PERIODONTITIS, ALVEOLA ABSCESS, NECROSIS, AND OTHER SEQUELÆ.

R. DENISON PEDLEY, M. R. C. S., L. D. S., ENG., F. R. C. S., EDIN.

When the upper teeth are affected, pus may find its way into the palate, or open into the floor of the nose, or into the antrum, as shown by the following case:

M. E., aged eight years. Measels three years ago, since which there has been a constant discharge from both ears. Five weeks ago left cheek began to ache and swell. A doctor removed a tooth and matter came away. A week later a lump was noticed below the left eye. The face being swollen and red, hot fomentations were applied. The patient was a well-nourished child, with fair hair and blue eyes. The face was considerably swollen on the left side, involving mostly the cheek, the latter being red and brawny. There was fluctuating swelling below and external to the inner canthus of the left eye, and at its summit pus was oozing from beneath a thick yellow crust. The crust being removed, examination with a probe showed that the fistulous opening was in connection with the antral cavity. On inspection of the mouth, a scarcely healed wound was seen between the temporary incisor and molar tooth, indicating clearly that the temporary canine had been removed. The hard palate on the effected side was quite normal, but, viewed from the outside, the anterior wall of the left antrum was more prominent than the right. No fluctation could be obtained through the antral wall. The child was placed under chloroform, and with an ordinary gimlet, previously rendered aseptic, the orifice through the canine socket was enlarged. The developing permanent canine, lying quite loose and in front of the opening, was removed. A probe was then thrust into the antrum and out on the cheek. The cavity of the antrum was carefully explored at the same time, the walls appearing healthy, except anteriorly. The sinus on the cheek was dressed with boracic ointment, and a pad placed over it. The antrum was syringed out with warm boracic lotion through the perforation of the jaw. The mother was directed to syringe it frequently with this lotion. The sinus of the cheek healed in a few days, and ten days later the wound in the mouth was closed up.

A collection of pus in the antrum in connection with temporary teeth is of rare occurrence. Owing to the small size of this cavity, the temporary teeth lie well outside of its walls, which are comparatively thick. In this case, pus from an alveolar abscess at the end of a temporary canine had burrowed deeply beneath the permanent canine, and, finding its way into the antrum, had pointed through the anterior wall and opened out on to the cheek. Far more serious injury is likely to occur as the result of periodontitis. Numerous cases are reported to Tomes* and Talter† where pyemia and death have occurred.

Mr. Arbuthnot Lane, of Guy's Hospital records the following case, an abstract of which is given :

*"Manual of Dental Surgery."

†"Surgery and Pathology."

### ALVEOLAR ABSCESS; PYEMIA; EXCISION OF THROMBOSED VEINS.

H. H., aet. four years. Suffering from a swelling over the left half of the lower jaw, which commenced with toothache about a week before. Five days previously an abscess burst into the mouth. Temperature on admission, 105.4 degrees. There was an alveolar abscess in connection with a second temporary molar. This was removed, and the abscess cavity scraped and thoroughfy cleansed out under an anesthetic. The boy became jaundiced, and suffered from recurring rigors. Mr. Lane excised the thrombosed veins, the external jugular and branches of the facial. The abscess cavity and bone were cleansed thoroughly and packed with iodoform gauze. Many inflamed lymphatic glands were removed. This operation was followed by disappearance of the jaundice, but subsequently the child died, and it was found there were a number of abscesses in the liver and lungs. These were produced by septic emboli before the operation took place. —*Lancet*, *Nov. 5th*, *1892*.

The case is instructive, not only in showing how pyemia may be caused by a tooth, but as showing that the removal of thrombosed veins is effectual in staying the pyemic process while yet local and uncomplicated.

A case very similar to that of Mr. Lane's was reported in *The Lancet* of June 25th, 1898, by Dr. Charles Bolton, the senior resident medical officer of the Evelina Hospital for Sick Children. On an abstract is given :

### A CASE OF ALVEOLAR ABSCESS; DEATH FROM PYEMIA IN EIGHT DAYS.

A boy, aged four years, brought to the Hospital on account of "pain in the sides." No previous illness except measles. For many weeks his mother had noticed that his teeth were "going very bad." She took no notice, as "bad teeth were so common." The boy made no complaint until five days ago, when he complained of "toothache."

His face was a little swollen on the right side on the next day, and a medical man who was sent for said patient had a gum boil, and ordered poultices. Two days later the mother took him to a hospital, as he seemed to be getting stupid, and here she was told that he had a gum-boil, and was given some medicine. The child was much worse on the following day, and complained of pain in the sides, and another medical man who was called in said that he had pleurisy, and ordered poultices to the chest. When the child was admitted, he was drowsey and fretful. Skin hot and dry; temperature, 104.6 degrees. There was a hard, tender, non-fluctuating swelling on the right side of the lower jaw, but nothing else abnormal could be felt in the neck. The breath was extremely offensive, and the tongue was coated with a greyish fur. The crowns of several teeth were completely decayed, and the second lower right temporary molar was carious and loose, a brownish purulent discharge issuing from its socket, the surrounding gum and adjacent part of the floor of the mouth being ulcerated.

The loose molar was extracted, and a quantity of thick pus escaped: The teeth and gums were thoroughly cleansed and the mouth and abscess cavity were irrigated every four hours with Sanitas lotion.

The child became steadily worse. The temperature ranged between 104 and 106 degrees, once or twice rising to 107 degrees. Mr. Makins exposed and removed most of the external jugular vein and branches of the facial. The child, however, died, and abscesses were found in both lungs. Some of Dr. Bolton's remarks concerning the dental aspect of the case are well worth recording: "The next point I wish to refer to is the inattention paid to children's teeth, not only by parents, but even by members of the medical profession. Of this there are numerous examples daily in the Evelina Hospital. It is quite a rule for these children never to have any pain at all. This point is not paid much attention to, but every one can verify it for himself, and he will then be surprised at the amount of destruction which can take place in children's teeth without the occurrence of any pain whatever. In reference to the treatment of alveola abscess in connection with temporary teeth, there is no doubt that in all cases the proper thing is to extract the tooth at once. Neither to apply poultices nor give medicine. The abscess then drains through the socket, the cause of the abscess is removed, and the permanent tooth which lies under the temporary one will probably be saved, which it may not be if the abscess is allowed to run its course unopened."

It is really a matter of surprise, considering the frequency of such septic inflammation, that fatal cases do not more frequently occur. It is by no means an uncommon occurrence in examining the mouths of children to find several sinuses or fistulæ in the mouth, each one connected with a separate tooth, duly discharging its share of pus, which is daily mixed with the food and saliva and swallowed. In connection wlth these teeth, or their roots, septic matter has escaped into the aveolus, periodontitis has followed; with the swelling, formation of pus, its escape through the alveolus, and the subsidence of the symptoms. Many such cases are of a passive type, passing through all the stages of inflammation and suppuration with very little pain and consequent distress, though it by no means follows that the results are less serious, as the following case will show:

A. B., a boy aet. five years, brought to the hospital because he had a sore mouth and bad breath. The mother stated that a few weeks ago the boy had a swelling on the side of his cheek.

The patient a puny child of dark complexion, had a foul breath and coughed incessantly. On examination, all the temporary molars were found deeply carious. On the right side in the upper jaw both upper molars were loose, the gum was dark red, and round the necks of the teeth there was a greyish slough. Pus was oozing out opposite the apex of the roots. On removing the teeth, the fangs were quite dark in color and necrosed. A good sized portion of the alveolus was found lying loose. This was removed, and also the crowns of two bicuspids which were offensive and almost black. The cavity was well mopped out with a solution of carbolic acid, 1 to 10. The mother was directed to syringe the cavity, and to wash the mouth out frequently with a solution of potassium permanganate. Internally, quinine and iron were prescribed.

This child had suffered very little pain indeed; so little that it was the child's breath alone which attracted attention.

Children who are wrongly fed, starved, or suffering from constitutional diseases, such as tuberculosis, rickets, or syphilis, are subjects of periodontitis. It generally commences with marginal ulceration of the gums, affecting carious and non-carious teeth alike. Many teeth may be involved, or one tooth alone. Sometimes it is symmetrical, as in the following case:

T. V., aged three years. A delicate boy with well-marked rickets. The mother says "food passes through him as soon as he eats it." The child cannot masticate because of loose teeth. On examination, the teeth are seen to be well formed, none are carious. The lower and upper canines are much raised above the teeth, and are somewhat loose. The patient cannot close his mouth properly, as only the canines meet. The gum is ulcerated round the necks of the teeth; the alveolus is expanded beneath the right lower, and above the right upper canine. Pus is oozing from sinuses. On removal of the teeth, the periosteal covering was found much thickened. The teeth, however, were neither carious nor discolored.

Exfoliation of the alveolar plates following periodontitis is of frequent occurrence after scarlet fever and measles, and though it is in many cases intimately connected with carious teeth and roots, this is not necessarily the case. When associated with carious teeth or necrotic roots, it would appear that the starting-point may be through the fang of a tooth or external to the teeth. The inflammation in the latter case spreads from a purulent gum margin which surrounds them. This latter condition is especially noticeable where the teeth are quite sound. The continuity of gum with periodontitis membrane, and the connection of the latter with the periosteum of the jaw, will serve to explain the means by which septic inflammation, either occurring within or outside, may involve the deeper tissues of the jaw; the symptoms in both cases are the same. The inflammation is of a passive type. A child is brought up for a swelling of the face, generally a foul breath and a discharge from the mouth, but very little pain. On examining the affected side, the gum will be found puffy, swollen, and deeply ulcerated round the necks of the teeth, leaving bare the alveolus. The teeth will be found quite loose, and pus oozing from the gum margins. Beyond removing loose and carious teeth, and seeing that the mouth is constantly cleaned, no treatment is required. In a month or six weeks the sequestrum will become quite loose, and can then be easily removed.—*British Journal of Dental Science.*

---

ARTICLE III.

